# Supplementary material for: How Much Is Enough? A Surgical Perspective on Imaging Modalities to Estimate Function and Volume of the Future Liver Remnant before Hepatic Resection
Source: Diagnostics (Basel). 2023 Aug 22;13(17):2726. doi: 10.3390/diagnostics13172726 (PMC10486462; doi:10.3390/diagnostics13172726)
Supplement: Supplementary file 1 [file diagnostics-13-02726-s001.zip › diagnostics-2534274-SI.pdf]

Table S1. List of parameters commonly acquired by Gd-EOB-DTPA MRI to evaluate liver functionality.

VARIABLE	HOW TO CALCULATE	AIM
*SIGNAL INTENSITY **(SI)***	$SI = \sqrt{S^2 + C_n^2}$, **S** is the true signal intensity (in the absence of noise) from tissue, and **Cn** is a noise-related constant.	SI is the measured signal intensity when averaged over time or a region of interest (ROI).
*APPARENT DIFFUSION COEFFICIENT **(ADC)***	**ADC = −ln (S/S0)/b** S0 is the signal intensity of no diffusion gradients and b is the b value of 0, 100, 300, 700, and 1000 s/mm2 along the three orthogonal main directions of the motion probing gradients.	Diffusion of water molecules in a particular ROI
*CONTRAST ENHANCEMENT RATIO **($CER_{HBP/TP}$)***	**$CER_{HBP/TP}$** = $[(SI_{HBP}-SI_{Pre})/(SI_{TP}-SI_{Pre})]$	The enhancement ratio between: SI in HBP contrast phase (30′ post-contrast), the transitional phase (TP, 3′ post-contrast), and precontrast T1 weighted images
*RELATIVE ENHANCEMENT **(RE_{HBP})***	**Re_{hbp}** = $(SI_{HBP}-SI_{Pre})/SI_{Pre}$	The contrast enhancement in terms of Signal Intensity (SI) between the HBP-contrast phase and the PRE-contrast.
REDUCTION RATE OF RELAXATION TIME	**Reduction rate (%)** = [(T1pre-T1post)/T1pre] x100	Relaxation time of the liver on T1 images before and after contrast administration
*LIVER TO SPLEEN RATIO **(LSR)***	**LSR** = SI_{20min}(liver)/SI_{20min} (spleen)	The ratio between SI of the liver and the spleen at 20′ after contrast injection
*LIVER TO MUSCLE RATIO **(LMR)***	**LMR** = SI_{20min}(liver)/SI_{20min} (muscle)	The ratio between SI of the liver and the muscle at 20′ after contrast injection
*HEPATOCELLULAR UPTAKE INDEX **(HUI)***	**HUI** = Liver volume x (SI_{20min}(liver)/SI_{20min}(spleen)-1	Amount of contrast uptake into hepatocytes measured on enhanced sequences.
*HEPATIC UPTAKE RATE **(K_{HEP})***	**K_{hep}** = 0.39/20 x [(1/$T1_{20min}$liver-1/$T1_{pre}$liver)/(1/$T1_{20min}$spleen-1/$T1_{pre}$spleen) − 0.77	Calculated from pharmacokinetic models of the liver and spleen.
